# Epidermal growth factor receptor (EGFR)—tyrosine kinase inhibitors as a first-line treatment for postoperative recurrent and EGFR-mutated non-small-cell lung cancer

**DOI:** 10.1093/icvts/ivab283

**Published:** 2021-10-15

**Authors:** Tetsuji Moriya, Masatsugu Hamaji, Akihiko Yoshizawa, Ryo Miyata, Misa Noguchi, Shigeyuki Tamari, Naohisa Chiba, Hideaki Miyamoto, Toshiya Toyazaki, Satona Tanaka, Yoshito Yamada, Yojiro Yutaka, Daisuke Nakajima, Akihiro Ohsumi, Toshi Menju, Hiroshi Date

**Affiliations:** 1 Department of Thoracic Surgery, Kyoto University Hospital, Kyoto, Japan; 2 Department of Diagnostic Pathology, Kyoto University Hospital, Kyoto, Japan

**Keywords:** Lung cancer, Epidermal growth factor receptor, Postoperative recurrence

## Abstract

**OBJECTIVES:**

To clarify survival outcomes and prognostic factors of patients receiving epidermal growth factor receptor (EGFR) - tyrosine kinase inhibitors (TKIs) as first-line treatment for postoperative recurrence.

**METHODS:**

A retrospective chart review was performed to identify consecutive patients who received EGFR-TKIs as first-line treatment for postoperative recurrence of non-small-cell lung cancer (NSCLC) harbouring EGFR gene mutations at our institution between August 2002 and October 2020. Therapeutic response, adverse events, progression-free survival (PFS) and overall survival (OS) were investigated. Survival outcomes were assessed using the Kaplan–Meier analysis. The Cox proportional hazards model was used for univariable and multivariable analyses.

**RESULTS:**

Sixty-four patients were included in the study. The objective response and disease control rates were 53% and 92%, respectively. Grade 3 or greater adverse events were noted in 4 (6.3%) patients, including 1 patient (1.6%) of interstitial pneumonia. The median follow-up period was 28.5 months (range 3–202 months). The total number of events was 43 for PFS and 23 for OS, respectively. The median PFS was 18 months, and the median OS was 61 months after EGFR-TKI treatment. In multivariable analysis, osimertinib showed a tendency to prolong PFS [hazard ratio (HR) 0.41, 95% confidence interval (CI) 0.12–1.1; *P* = 0.071], whereas the micropapillary component was significantly associated with shorter OS (HR 2.1, 95% CI 1.02–6.9; *P* = 0.045).

**CONCLUSIONS:**

EGFR-TKIs as first-line treatment appeared to be a reasonable treatment option in selected patients with postoperative recurrent EGFR-mutated NSCLC. Osimertinib and the micropapillary component may be prognostic factors.

**Figure:**
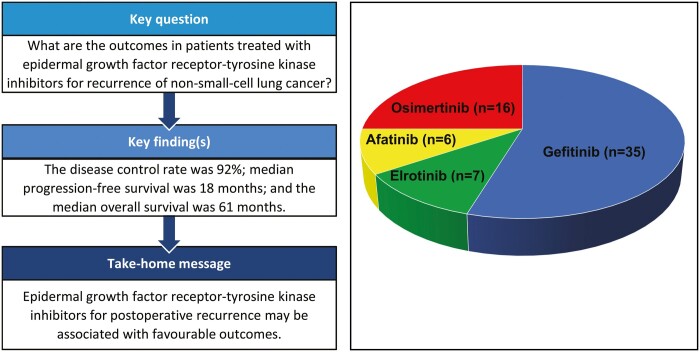

## INTRODUCTION

Epidermal growth factor receptor (EGFR) mutations are well-established oncogenic targets for management of advanced-stage non-small-cell lung cancer (NSCLC) [1, 2]. According to the guidelines provided by the Japan Lung Cancer Society, European Society of Medical Oncology (ESMO) and National Comprehensive Cancer Network (NCCN), EGFR-tyrosine kinase inhibitors (TKIs) are recommended as the first-line treatment for stage IV NSCLC harbouring EGFR gene mutations, whereas there is a dearth of data on postoperative recurrent stage IV patients in practice-based settings [3–6].

Specifically, recent clinical trials for patients with advanced and EGFR-mutated NSCLC demonstrated objective response rates ranging from 63% to 80%, disease control rates of >90% and grade 3 or greater adverse events ranging from 9% to 50% as short-term outcomes [6–11]. Regarding long-term survival outcomes in those trials, median progression-free survival (PFS) ranged from 8 to 11 months in patients receiving first- or second-generation EGFR-TKIs and >18 months in those receiving third-generation EGFR-TKIs as first-line therapy [7–10]. However, it is unknown whether recommendations from the above guidelines and findings from these trials can be translated into patients with postoperative recurrence because patients with postoperative recurrence may be older, more morbid, associated with a more complicated history of illness and poorer cardiopulmonary function than the patients enrolled in the clinical trials [6, 12].

The aim of this study was to investigate the short-term and long-term outcomes and management of patients receiving first-line EGFR-TKI for postoperative recurrence of NSCLC harbouring EGFR gene mutations and to elucidate potential prognostic factors associated with favourable (or poor) survival outcomes in these patients.

## PATIENTS AND METHODS

This study was approved by the Institutional Review Board of Kyoto University Hospital (reference number: R2504, approval date: 22 June 2020). The requirement for informed patient consent was waived. This retrospective observational study was performed according to the Strengthening the Reporting of Observational Studies in Epidemiology (STROBE) Statement (Supplementary Material, Table S1) [13]. This study was performed in accordance with the Declaration of Helsinki (1975, as revised in 2013) and Good Clinical Practice guidelines (as defined by the International Conference on Harmonization).

A retrospective chart review was performed to identify consecutive patients who received EGFR-TKIs as first-line treatment for postoperative recurrence of NSCLC harbouring EGFR gene mutations at our hospital between August 2002 and October 2020. The patients must be within grade 0–2 on the Eastern Cooperative Oncology Group (ECOG) scale of performance status at the beginning of medication. We excluded patients who had any distant metastasis at the initial surgery and those who received cytotoxic chemotherapy as a first-line treatment for postoperative recurrence.

Pathological staging was classified according to the International System for Staging Lung Cancer by the eighth edition. NSCLC histology was classified according to World Health Organization classification. EGFR gene mutations in exons 18, 19, 20 and 21 were analysed in resected specimens at the initial surgery.

### Management of epidermal growth factor receptor-tyrosine kinase inhibitors and follow-up

Types of EGFR-TKIs (first-, second- or third-generation) were selected after discussions at the multidisciplinary thoracic oncology board of our hospital. Gefitinib was usually administered at a dose of 250 mg/day daily, erlotinib at a dose of 150 mg/day daily, afatinib at a dose of 40 mg/day daily and osimertinib at a dose of 80 mg/day daily. The dose was reduced or discontinued at the attending physician’s discretion regarding toxicity. Chest and abdominal computed tomography (CT) scans were typically obtained within 3–6 months after the introduction of TKIs for the assessment of efficacy. Outpatient follow-up was generally set up every 1–3 months. Brain magnetic resonance imaging was obtained on an as-needed basis, although it was not routinely obtained if patients were asymptomatic. When progressive disease (PD) was suspected, additional examinations were scheduled, including re-biopsy (to examine T790M mutation) and positron emission tomography.

### Short-term and long-term outcomes

In this study, the therapeutic response was assessed using the Response Evaluation Criteria in Solid Tumors (RECIST) and was recorded based on the CT scan obtained within 3–6 months of initiating EGFR-TKIs. The response rate was the sum of the complete response and partial response. The disease control rate was the sum of complete response, partial response and stable disease. Adverse events were evaluated according to the Common Terminology Criteria for Adverse Events (CTCAE). PFS was calculated from EGFR-TKI until the date of PD or death, or until the date the patient was last known to be alive and progression-free. Overall survival (OS) was calculated from EGFR-TKI until the date of death from any cause or until the date on which the patient was last known to be alive. The last follow-up date was 15 April 2021.

### Statistical analysis

Based on the statistical and data-reporting guidelines of the European Journal of Cardiothoracic Surgery and Interactive Cardiovascular and Thoracic Surgery [14], descriptive statistics were reported as frequencies and percentages for categorical variables, and as medians and interquartile ranges for continuous variables. The collected data included age, sex, operative procedures, extent of resection, pathological stage in the eighth edition, tumour histology and histopathological subtypes, type of EGFR gene mutation, type of EGFR-TKIs used, site and number of recurrent lesions, adverse effects, disease-free interval and follow-up information until death or April 2021. We did not attempt to replace missing data and included all patients in the analyses using the available data. Survival curves were estimated using the Kaplan–Meier method. The Cox proportional hazards model was used for univariable and multivariable analyses, and the hazard ratios (HRs) were calculated. If a *P*-value was <0.15 on univariable analysis, it was included in the multivariable analysis. We did not correct for multiple testing in any of the tests performed. When the *P*-value was <0.05, it was considered statistically significant. All comparisons were two-sided. All statistical analyses were performed using JMP software (version 15.2; SAS Institute, Cary, NC, USA).

## RESULTS

Sixty-four patients were included in this study. The median age was 70.5 years and 29 (45.3%) patients were male. The characteristics of the 64 eligible patients are shown in Table 1.

**Table 1:** Characteristics of the 64 patients who received EGFR-TKIs as a first-line therapy for postoperative recurrent and EGFR-mutated NSCLC

Variables	
Age (years), median (interquartile range)	70.5 (66–77)
Sex, *n* (%)	
Male	29 (45)
Female	35 (55)
Smoking history, *n* (%)	
Never smoker	32 (50)
Previous smoker	22 (34.3)
Current smoker	9 (14.1)
Unknown	1 (1.6)
Completeness of resection, *n* (%)	
R0 resection	62 (97)
R1 resection	2 (3)
Extent of resection, *n* (%)	
Lobectomy	54 (84)
Sublobar resection	10 (16)
Pathological stage at initial surgery (TNM 8th edition), *n* (%)	
Stage 1A	18 (28.1)
Stage 1B	7 (11)
Stage 2A	5 (8)
Stage 2B	9 (14.1)
Stage 3A	24 (37.5)
Stage 3B	1 (1.6)
Histology, *n* (%)	
Adenocarcinoma	62 (97)
Adenosquamous cell carcinoma	2 (3)
Any micropapillary component, *n* (%)	35 (55)
Micropapillary-predominant	4 (6.3)
Any solid component	21 (33)
Solid predominant	4 (6.3)
EGFR gene mutations, *n* (%)	
Exon 21 point mutation	32 (50)
Exon 19 deletion	27 (42.2)
Exon 18 point mutation	4 (6.3)
Exon 20 point mutation	1 (1.6)
PD-L1 protein expression in the primary lesion, *n* (%)	
≥50% positive	4 (6.3)
1–49% positive	14 (21.9)
Negative	7 (11)
Unknown	39 (61)
Recurrence lesions, *n* (%)	
Single lesion only	34 (53)
Multiple lesions	30 (47)
Any brain metastasis	8 (13)
EGFR-TKIs, *n* (%)	
Gefitinib (first-generation)	35 (55)
Erlotinib (second-generation)	7 (11)
Afatinib (second-generation)	6 (9)
Osimertinib (third-generation)	16 (25)
DFI (months), median (interquartile range)	15.5 (9–29)

DFI: disease-free interval; EGFR: epidermal growth factor receptor; NSCLC: non-small-cell lung cancer; PD-L1: programmed death-ligand 1; TKI: tyrosine kinase inhibitor; TNM: tumour, node and metastasis.

### Responses to epidermal growth factor receptor-tyrosine kinase inhibitors and adverse events

Complete response and partial response were achieved in 3 (4.7%) and 31 (48.4%) patients, respectively. Stable disease was obtained in 25 patients (39.1%). The objective response rate was 53% and the disease control rate was 92%.

Adverse events in this study were summarized in Table 2. Any adverse event was noted in 57 patients (89%). Grade 3 or greater adverse events were confirmed in 4 patients (6.3%), including interstitial pneumonitis (*n* = 1), gastrointestinal bleeding (*n* = 1), thrombocytopaenia (*n* = 1) and neutropenia (*n* = 1). The patient with interstitial pneumonia was treated with steroid pulse therapy and recovered steadily. Reduction in dosage or discontinuation of the first-line EGFR-TKI within the first 6 months due to side effects was noted in 31 (48.4%) patients, including 7 (10.9%) patients who switched to another EGFR-TKI.

**Table 2:** Adverse events in association with EGFR-TKIs as a first-line treatment in this study

	Grade 1 (*n* = 31), *n* (%)	Grade 2 (*n* = 34), *n* (%)	Grade 3 (*n* = 4), *n* (%)
Rash or acne	16 (25)	18 (28.1)	
Diarrhoea	2 (3.1)	8 (12.5)	
Elevation of hepatic transaminases	5 (7.8)	4 (6.3)	
Paronychia	2 (3.1)	3 (4.7)	
Nausea	2 (3.1)		
Thrombocytopaenia			1 (1.6)
Neutropenia			1 (1.6)
Gastrointestinal bleeding			1 (1.6)
Interstitial pneumonitis			1 (1.6)
Haemoptysis		1 (1.6)	
Malaise	1 (1.6)		
Haematuria	1 (1.6)		
Fever	1 (1.6)		
Epistaxis	1 (1.6)		

### Survival outcomes

**Figure 1:**
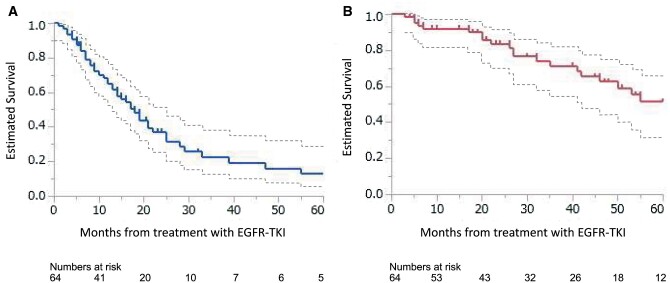
The estimated progression-free survival following EGFR-TKI in all 64 patients (**A**). The estimated overall survival following EGFR-TKI in all 64 patients (**B**). The dotted lines indicate 95% confidence intervals. EGFR-TKI: epidermal growth factor receptor-tyrosine kinase inhibitor.

**Figure 2:**
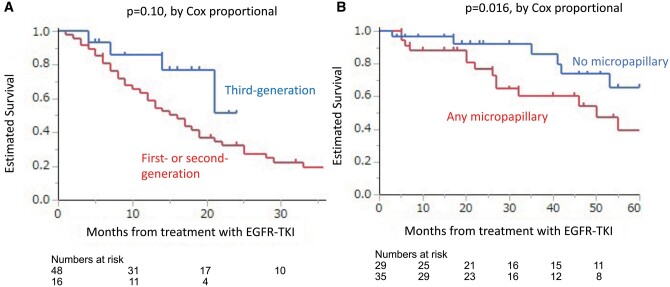
Comparison of the estimated progression-free survival following EGFR-TKI in patients receiving third-generation TKI (blue line) and patients receiving first- or second-generation TKI (red line) (**A**). Comparison of the estimated overall survival following EGFR-TKI in patients with micropapillary components (red line) and patients without micropapillary components (blue line) (**B**). EGFR-TKI: epidermal growth factor receptor-tyrosine kinase inhibitor.

The median follow-up period was 28.5 months (range 3–202 months). The total number of events was 43 for PFS and 23 for OS, respectively. The median PFS and OS of all patients after the initiation of EGFR-TKIs were 18 and 61 months, respectively. The 3- and 5-year PFS rates were 22.5% and 12.9%, respectively (Fig. 1A), and the 3- and 5-year OS rates were 71.3% and 51.5%, respectively (Fig. 1B). Univariable and multivariable analyses of potentially relevant factors associated with PFS and OS were shown in Tables 3 and 4, respectively. In univariable analysis, the disease-free interval [HR 0.20, 95% confidence interval (CI) 0.02–1.06; *P* = 0.060], no solid component (HR 0.61, 95% CI 0.32–1.1; *P* = 0.12) and third-generation TKI versus first- or second-generation TKI (HR 0.41, 95% CI 0.15–1.2; *P* = 0.10) showed tendencies to prolong PFS (Fig. 2A); in multivariable analysis, third-generation TKI versus first- or second-generation TKI (HR 0.41, 95% CI 0.12–1.1; *P* = 0.071) showed tendencies to prolong PFS. In univariable analysis, the micropapillary component (HR 2.9, 95% CI 1.2–7.5; *P* = 0.016) was a statistically significant factor associated with shorter OS (Fig. 2B), and disease-free interval (HR 0.06, 95% CI 0.001–1.35; *P* = 0.12) and pathological stage III versus stage I or II at initial surgery (HR 2.1, 95% CI 0.88–5.2; *P* = 0.091) showed a tendency to shorten OS. In multivariable analysis, the micropapillary component (HR 2.1, 95% CI 1.02–6.9; *P* = 0.045) was a statistically significant factor associated with shorter OS.

**Table 3:** Cox proportional hazards models for progression-free survival in univariable and multivariable analyses

Variables		Univariable		Multivariable	
		HR (95% CI)	*P*-value	HR (95% CI)	*P*-value
Age	Every 1 year	1.38 (0.28–7.4)	0.69		
Gender	Male/female	1.22 (0.66–2.2)	0.52		
Disease-free interval	Every 1 month	0.20 (0.02–1.06)	0.060	0.40 (0.046–2.5)	0.37
Pathological stage at initial surgery	3/1 or 2	1.63 (0.89–2.9)	0.11	1.5 (0.79–2.7)	0.22
No solid component	No/yes	0.61 (0.32–1.1)	0.12	0.64 (0.33–1.3)	0.20
Recurrent lesions	Multiple/single	0.89 (0.50–1.6)	0.70		
Types of EGFR mutations	Exon 19/Exon 21	1.16 (0.62–2.2)	0.65		
TKI generation	Third/first or second	0.41 (0.15–1.2)	0.10	0.41 (0.12–1.1)	0.071

CI: confidence interval; EGFR: epidermal growth factor receptor; HR: hazard ratio; TKI: tyrosine kinase inhibitor.

**Table 4:** Cox proportional hazard models for overall survival in univariable and multivariable analyses

Variables		Univariable		Multivariable	
		HR (95% CI)	*P*-value	HR (95% CI)	*P*-value
Age	Every 1 year	1.3 (0.14–14.8)	0.83		
Gender	Male/female	0.91 (0.40–2.1)	0.82		
Disease-free interval	Every 1 month	0.06 (0.001–1.4)	0.12	0.12 (0.002–2.5)	0.22
Pathological stage at initial surgery	3/1 or 2	2.1 (0.88–5.2)	0.091	1.5 (0.61–3.8)	0.37
Micropapillary component	Yes/no	2.9 (1.2–7.5)	0.016	2.1 (1.0–6.9)	0.045
Recurrent lesions	Multiple/single	1.8 (0.76–4.0)	0.17		
Types of EGFR mutations	Exon 19/Exon 21	1.5 (0.63–3.5)	0.37		
TKI generation	Third/first or second	0.54 (0.07–4.4)	0.57		
No solid component	No/yes	0.62 (0.27–1.5)	0.27		

CI: confidence interval; EGFR: epidermal growth factor receptor; HR: hazard ratio; TKI: tyrosine kinase inhibitor.

### Management of progressive disease after first-line epidermal growth factor receptor-tyrosine kinase inhibitor

During the follow-up period, PD was observed in 39 patients. Among them, 21 (53.8%) patients switched to another TKI, 10 (25.6%) patients underwent cytotoxic chemotherapy (3 patients received single-agent pemetrexed, 3 patients received single-agent docetaxel, 2 patients received carboplatin, paclitaxel, bevacizumab and atezolizumab, 1 patient received carboplatin and nab-paclitaxel and 1 patient received carboplatin, pemetrexed and bevacizumab), 2 patients underwent salvage surgery (for pulmonary metastasis in both patients) and 2 patients received palliative radiotherapy only. The association between first-line EGFR-TKI and second-line management is summarized in Table 5, with third-generation TKI (osimertinib) being the most common TKI as second-line TKI in 13 patients. Re-biopsy to confirm T790M mutation was typically performed before switching to osimertinib after 2015.

**Table 5:** Association between EGFR-TKIs as first-line therapy and the second-line management in patients with progressive disease after first-line therapy (*n* = 39)

		Second-line management (*n* = 39)							
		Gefitinib (*n* = 1)	Erlotinib (*n* = 6)	Afatinib (*n* = 1)	Osimertinib (*n* = 13)	Cytotoxic chemo (*n* = 10)	Salvage surgery (*n* = 2)	Palliative RT (*n* = 2)	BSC (*n* = 4)
First-line therapy (*n* = 39)	Gefitinib (*n* = 24)		6		9	5	1	1	2
	Erlotinib (*n* = 6)			1	1	1		1	2
	Afatinib (*n* = 6)	1			3	1	1		
	Osimertinib (*n* = 3)					3			

BSC: best supportive care; chemo: chemotherapy; EGFR: epidermal growth factor receptor; RT: radiotherapy; TKI: tyrosine kinase inhibitor.

After the introduction of osimertinib as a first-line EGFR-TKI, 3 patients were found to have PD. Two of these received carboplatin, paclitaxel, bevacizumab and atezolizumab as second-line therapy, and the other patient received carboplatin and nab-paclitaxel.

## DISCUSSION

After curative or complete resection of NSCLC, 30–40% are known to recur [15, 16]; however, there have been few studies on the management or treatment of patients with postoperative recurrence of resected NSCLC, presumably because follow-up policies of postoperative patients vary according to the institution and country [17]. Treatments for recurrent NSCLCs, a majority of which are distant lesions, appear to prolong survival in some patients [15, 16]. Although EGFR gene status is not routinely examined in surgically resected specimens, it is worthwhile to examine any EGFR gene mutations in the specimens because EGFR-TKIs may extend survival in patients with postoperative recurrence of EGFR-mutated lung adenocarcinoma [18].

Current recommendations of the guidelines derived from previous randomized controlled trials examining the effect of EGFR-TKIs on advanced-stage patients without separating newly diagnosed and postoperative recurrent NSCLCs [7, 19, 20]. Specifically, data from the FLAURA trial appeared to be the most updated on recent patients receiving EGFR-TKIs for advanced and EGFR-mutated NSCLC. However, there are several challenges in translating the data into patients with postoperative recurrence in a practice-based setting. There was no description of the number of patients with postoperative recurrence enrolled in the study. It should also be noted that patient characteristics in the FLAURA trial may differ from those in real-world data. In our study, compared to the FLAURA trial, patients tended to be older, included more males and more Asian patients, and included fewer patients with brain metastasis.

Our major findings were that osimertinib, a third-generation EGFR-TKI, compared to first- and second-generation TKIs, showed a tendency to extend PFS and that the micropapillary component was significantly associated with shorter OS after recurrence. The former finding was consistent with those from the FLAURA trial, and the latter may be a novel finding for patients with EGFR-mutated NSCLC.

Very few data are available on the association between adenocarcinoma subtypes and responses to EGFR-TKIs [21]. Previous studies suggested that the micropapillary component as a histological subtype appeared to be associated with postoperative recurrence in patients with EGFR-mutated lung adenocarcinoma [22, 23]. It was largely unknown whether micropapillary components may predict the response of recurrent lesions to EGFR-TKIs in these patients. Interestingly, our findings suggested that EGFR-mutated adenocarcinoma with micropapillary components may not respond to treatment with EGFR-TKIs. It was previously not demonstrated because relatively small specimens obtained by transbronchial or CT-guided biopsy may theoretically be less sensitive in identifying micropapillary components than surgical specimens. We emphasize that the findings are preliminary and require further studies because the histology was obtained at the time of initial pulmonary resection and not at recurrence in a majority of patients.

Management of PDs after EGFR-TKI therapy may play an important role in maintaining the quality of life or extending survival for patients receiving EGFR-TKIs. In this study, 39 (60.9%) patients had PD after EGFR-TKI, and a majority of them underwent treatments for PDs, which ranged from salvage surgery to cytotoxic chemotherapy, with switching to another EGFR-TKI such as osimertinib being the most frequent pattern, where re-biopsy to examine T790M mutation plays an important role.

In 2015, we started re-biopsy to examine T790M mutation before switching to osimertinib in 2015, as recommended in the guidelines from Japanese Lung Cancer Society. Before 2015, no patient switched from first-generation or second-generation EGFR-TKIs to osimertinib (third-generation EGFR-TKI) or benefitted from osimertinib as second-line therapy for PD after first-line EGFR-TKI.

Osimertinib is a third-generation EGFR-TKI, and it remains unknown what the best management is for PD after first-line osimertinib. Our treatment of choice was cytotoxic chemotherapy with or without immune checkpoint inhibitors, whereas our data are too immature to estimate long-term outcomes. Currently, an increasing number of patients appear to have received osimertinib, rather than first- or second-generation EGFR-TKIs, the management of PD after osimertinib would be the next challenge of interest. Nivolumab, which is an immune checkpoint inhibitor, may be a treatment of choice for disease progression after EGFR-TKI in selected patients [24, 25]; however, the median PFS was from 1 to 2 months, and more data are required to identify patient groups that benefit from immune checkpoint inhibitors after EGFR-TKIs.

### Limitations

The limitations of our study include its retrospective design, single-centre experience and sample size in a relatively large time frame. Selection of first-line or second-line EGFR-TKI was subject to physician's preference; therefore, selection bias, which is inevitable due to the study design, could be another limitation. The diagnosis of postoperative recurrence was typically made based on radiological findings and not always on biopsies. Our patient cohort ranged from 2002 to 2020 at the time of initiating EGFR-TKIs, and several changes in management should have occurred. Especially at the beginning of our series, indications for EGFR-TKIs were not established from the guidelines. Specifically, it was not until 2014 that the guidelines from Japanese Lung Cancer Society recommended EGFR-TKI over cytotoxic chemotherapy as first-line therapy for patients with EGFR-mutated advanced NSCLC, in alignment with the guidelines from ESMO and NCCN.

## CONCLUSION

In conclusion, our findings suggested that treatment with third-generation EGFR-TKI had a tendency to extend PFS in patients with postoperative recurrence of EGFR-mutated NSCLC. Micropapillary components may be a prognostic factor associated with poor OS in these patients. A multi-institutional study with a larger sample size is required to confirm our findings and is now under way. A prospective study, which enables us to perform more rigorous analyses, would be the following step.

## SUPPLEMENTARY MATERIAL

Supplementary material is available at *ICVTS* online.

### Funding

No funding.

**Conflict** **of interest:** none declared.

### Author contributions

**Tetsuji Moriya**: Conceptualization; Data curation; Formal analysis; Investigation; Methodology; Validation; Writing—original draft; Writing—review & editing. **Masatsugu Hamaji:** Conceptualization; Data curation; Formal analysis; Investigation; Methodology; Resources; Writing—original draft; Writing—review & editing. **Akihiko Yoshizawa:** Conceptualization; Data curation; Investigation; Methodology; Writing—review & editing. **Ryo Miyata:** Conceptualization; Data curation; Investigation; Methodology; Writing—review & editing. **Misa Noguchi:** Data curation; Investigation; Methodology; Writing—review & editing. **Shigeyuki Tamari:** Conceptualization; Data curation; Investigation; Methodology; Writing—review & editing. **Naohisa Chiba:** Conceptualization; Data curation; Investigation; Methodology; Writing—review & editing. **Hideaki Miyamoto:** Conceptualization; Data curation; Investigation; Methodology; Writing—review & editing. **Toshiya Toyazaki:** Conceptualization; Data curation; Investigation; Methodology; Writing—review & editing. **Satona Tanaka:** Conceptualization; Data curation; Investigation; Methodology; Writing—review & editing. **Yoshito Yamada:** Conceptualization; Data curation; Investigation; Methodology. **Yojiro Yutaka:** Conceptualization; Data curation; Investigation; Methodology; Writing—review & editing. **Daisuke Nakajima:** Data curation; Formal analysis; Investigation; Methodology; Writing—review & editing. **Akihiro Ohsumi:** Conceptualization; Data curation; Investigation; Methodology; Writing—review & editing. **Toshi Menju:** Conceptualization; Data curation; Investigation; Methodology; Writing—review & editing. **Hiroshi Date:** Conceptualization; Data curation; Investigation; Methodology; Project administration; Supervision; Writing—review & editing.

### Reviewer information

Interactive CardioVascular and Thoracic Surgery thanks Mohsen Ibrahim, Mohammad Behgam Shadmehr and the other, anonymous reviewer(s) for their contribution to the peer review process of this article.

## ABBREVIATIONS

CI: Confidence interval
CT: Computed tomography
EGFR: Epidermal growth factor receptor
HR: Hazard ratio
NSCLC: Non-small-cell lung cancer
OS: Overall survival
PD: Progressive disease
PFS: Progression-free survival
TKI: Tyrosine kinase inhibitor
